# Effect and validation of plate thickness dimensional effects on grouting compactness in bridge ducts based on ultrasonic testing

**DOI:** 10.1371/journal.pone.0328775

**Published:** 2025-08-14

**Authors:** Chao Feng, Miren Rong, Yinping Pang, Hailong Wang, Lanxin Luo, Zhikun Xue, Ying Yuan, Dongyang Geng, Jiayin Li

**Affiliations:** 1 School of Urban Geology and Engineering, Hebei GEO University, Shijiazhuang, China; 2 Hebei Technology Innovation Center for Intelligent Development and Control of Underground Built Environment, Shijiazhuang, China; 3 Shijiazhuang University of Applied Technology, Shijiazhuang, China; 4 Shijiazhuang Tiedao University, Shijiazhuang, China; 5 Hebei Academy of Building Research Co., Ltd, Shijiazhuang, China; Shandong University of Technology, CHINA

## Abstract

Based on the theory of ultrasonic non-destructive testing, this study investigates the dimensional effect of plate thickness on the detection of grouting compactness in prestressed bridge ducts. By combining finite element simulation with engineering case validation, a high-precision time-domain analysis model is proposed to qualitatively and quantitatively evaluate the grouting compactness of prestressed bridge pipeline with varying plate thicknesses. This study employs ABAQUS finite element software to simulate and analyze the excitation, propagation, and reception characteristics of ultrasonic waves in concrete structural components. A time-domain analytical theoretical model is established to evaluate the detection results of pipelines with various defects. By comparing the simulation results of the theoretical model with actual engineering detection cases, the time-domain analytical theoretical model demonstrates high feasibility and reliability. The results indicate that the time-domain analysis method achieves high accuracy when utilizing the correlation between plate thickness and the first wave acoustic time. This approach enables both qualitative and quantitative analysis of pipeline density using ultrasonic non-destructive testing, thereby improving the accuracy and efficiency of damage assessment for prestressed pipe grouting compactness.

## 1. Introduction

China is a global leader and the largest market in high-speed rail technology and infrastructure. In recent years, China has witnessed rapid development in its expressway and railway infrastructure. As of 2024, the total operational mileage of high-speed railways nationwide has exceeded 48,000 kilometers (China State Railway Group Annual Report), with large-span prestressed concrete beam bridges accounting for over 60% of these structures. With the “Transport Power Construction Outline” mandating a 100-year service life requirement for infrastructure, ensuring the long-term performance of large-span prestressed concrete beam bridges has emerged as a critical focus in engineering practice. These bridges incorporate prestressed ducts. Following the tensioning of prestressed steel strands, immediate grout injection into the ducts is required to form a monolithic load-bearing structure [1–4]. However, poor grout densification in the ducts seriously affects the overall safety and durability of the bridge [5,6]. Therefore, the issue of duct densification has attracted widespread attention in both academic research and engineering applications. The methods for detecting duct densification can be classified into destructive and non-destructive testing methods. Although some studies suggest that the surface condition of the concrete duct or small samples taken may not accurately represent the overall structure, and non-destructive testing might lead to misleading results [7], traditional destructive testing methods are more costly, complex to operate, damage the internal prestressed structure, and are inefficient [8]. Non-destructive testing has become the research focus due to its flexibility, non-damaging nature, and the possibility of employing multiple methods simultaneously [9]. Conventional non-destructive testing methods for evaluating the grout densification in prestressed ducts of bridge structures include rebound methods, radar methods, infrared thermography, impact echo methods, and ultrasonic methods [10–13].

The rebound method is based on the surface hardness of concrete, where the rebound value is directly measured and then converted into the concrete’s strength value using a strength calibration curve. The estimated concrete strength of the component is derived based on a 95% confidence level. However, this method overlooks the difference in strength between the internal and surface concrete, making it difficult to assess the overall quality of the component [14]. Janku et al. [15] compared several methods and found that infrared thermography (IRT) was particularly successful in detecting voids near the surface of concrete bridges, although it has certain temperature requirements for the testing site. Ground-penetrating radar (GPR) and ultrasonic pulse echo (UPE) methods provided better results when detecting deeper internal voids within concrete bridges compared to IRT. The impact-echo technique employs a hardened steel impactor to generate broadband stress waves through controlled surface impact. These waves propagate through the test structure and are detected by a piezoelectric receiver, which acquires time-domain waveforms for subsequent fast Fourier transform (FFT) analysis to obtain frequency-domain response spectra. The internal defects’ size and location of the object can be inferred from the signal changes in the frequency curve [16]. Liu et al. [17] successfully used the impact echo method to detect the grouting density of concrete hollow slabs, although the results also indicated that the method has certain limitations in terms of its dependence on material properties and the interpretability of test results. Yang et al. [18]pointed out that the impact echo method is effective in detecting the location and depth of defects, but the accuracy of the results is influenced by the material of the pipes. Additionally, when the critical surface of the defect is perpendicular to the detection surface, the detection becomes more difficult, and errors in defect depth measurements are more noticeable. Detection accuracy is higher for metal corrugated tubing compared to PVC pipes. Li and Long [19] verified the feasibility and practicality of using HHT-based testing methods to assess the grouting quality of plastic pipes through on-site IE testing. However, this method is relatively complex and requires further experimentation to validate its accuracy. Ultrasonic testing functions similarly to impact-echo techniques by generating stress waves from the surface of the test object. Compared to impact methods, ultrasonic transducers deliver more stable signals, enable multi-point excitation, and offer higher frequencies with stronger penetration performance. This method is capable of emitting predefined waveforms and recording reflected signals after propagation through the medium, enabling the collection of significantly more information [20]. In heterogeneous media, variations in relevant parameters can indicate internal damage to the detected object.

To date, numerous techniques have been proposed for structural health monitoring (SHM) and non-destructive testing (NDT) of concrete structures [21]. Among various methods, ultrasonic testing stands out as one of the most promising approaches [22]. Ultrasonic stress pulses are designed to induce high-frequency mechanical stress waves, encompassing both impact-echo and ultrasonic echo testing methodologies [23–27]. Many scholars have conducted extensive research on ultrasonic testing of the grouting density in prestressed ducts. Kou et al. [28] proposed a laser-based, non-contact nonlinear ultrasonic testing method. This method uses nonlinear numerical simulation with the finite element method (FEM) to model the higher harmonics generated by closed surface cracks. Simulation results indicate that the acoustic nonlinearity parameter increases with the length of micro-closed cracks but decreases as their depth increases. Experimental validation further confirms the effectiveness of this method in defect detection. Korolkov et al. [29] derived the density expressions for materials under both shear and longitudinal wave conditions based on the propagation velocity equations of ultrasonic waves in solid media. They also identified factors influencing the measurement of ultrasonic wave speed. Hsiao [30] conducted a numerical analysis on the transient response of dense concrete blocks and defect-containing concrete blocks under impact loading. The results indicated that the impact response of the concrete blocks was related to their internal vibration modes, with the longitudinal wave speed determining the peak frequency. Wang et al. [31] employed the AGI-BWG grouting quality detection system to verify the ultrasonic method for evaluating the grouting quality in prestressed ducts. Their results showed that the ultrasonic method reflects the grouting quality in prestressed ducts to some extent, with the evaluation index being the propagation velocity of elastic waves within the ducts. Sun and Zhu [32] proposed an ultrasonic guided wave method using tie rods as waveguides to detect honeycombing and voids around the rods. Experiments were conducted on three different bonding conditions (solid, honeycombed, and voided) of tie rods at different stages (initial, 7 days, and 110 days), successfully identifying internal defects in concrete and evaluating the bonding conditions of the tie rods with concrete at different times. Kuchipudi et al. [33] used the Half-Skip Total Focusing Method (HSTFM) to process target scattering signals reflected from the back wall of concrete and imaged vertical cracks within the concrete. They found that the HSTFM-based analysis could more accurately determine the depth and shape of the cracks compared to SAFT/TFM, and it required less computational cost than methods like Reverse Time Migration (RTM). Callejas et al. [34] conducted a comprehensive ultrasonic scanning of multiple Concrete-Filled Steel Tubes (CFST) samples with different air void ratios. Results revealed a strong positive correlation between the average Backscatter Ultrasonic Attenuation (BUA) value within the CFST window and the void percentage (Pearson correlation coefficient r = 0.9873). The BUA algorithm proved effective in identifying defect regions within the CFST. Similarly, frequency-domain analyses using Fast Fourier Transform (FFT) and Short-Time Fourier Transform (STFT) proved to be highly sensitive in detecting internal damage, with Pearson correlation coefficients of r = −0.9799 and r = −0.9672, respectively. Dinh et al. [35] employed one of the most advanced ultrasonic shear wave devices (MIRA 3D) to collect data from concrete samples with different embedded defects. Data visualization was performed using the Synthetic Aperture Focusing Technique (SAFT). Comparative analysis of the reconstructed images indicates that ultrasonic shear wave tomography ranks among the most effective methods for imaging concrete structures. Mutlib et al. [36] utilized Short-Time Fourier Transform (STFT) to effectively identify damages of different sizes based on graphical interpretation. Simulation conducted with COMSOL software confirmed the effectiveness of the detection method. The Total Focusing Method (TFM), using a linear ultrasonic array with a single emitter and multiple receivers, allows for more aperture data to be acquired with the same number of probes. This method effectively averages and suppresses random noise in single echo signals, thereby improving imaging quality and detection sensitivity by leveraging the advantages of the algorithm [37,38]. Yang et al. [39] pointed out that the Total Focusing Method (TFM) is prone to missing specific defects in blind spots located farther from the sensor or at larger deflection angles. They proposed the Diffusion Attenuation Compensation Method (DACM), Solid Directional Correction Method (SDCM), and the Diffusion Attenuation Compensation-based Solid Directional Correction Method (DAC-SDCM). Experimental results showed that DACM is suitable for detecting defects located at greater distances, SDCM can detect defects at larger deflection angles, and DAC-SDCM significantly improves detection performance for defects at greater distances and with larger deflection angles. These methods can significantly enhance the detection capability of defects in blind spots. Several scholars have reviewed the latest autonomous ultrasonic non-destructive testing technologies realized by deep learning methods, identifying opportunities and directions for current research, including data denoising, image interpretation, uncertainty quantification, and autonomous awareness in automated systems, aiming to provide a roadmap for future research and development in this field [40]. Subsequently, Kuchipudi and Ghosh [41] proposed a region-based Convolutional Neural Network (CNN) capable of automatically detecting, locating, and segmenting features corresponding to defects in noisy ultrasonic images with multiple characteristics. The trained model’s predictions for the test dataset can detect and mask pixels corresponding to defects in the ultrasonic images, with an optimal mean Average Precision (mAP) of 0.98. The tests revealed that the developed model’s segmentation results outperformed other state-of-the-art defect detection networks. To sum up, a lot of research achievements have been made on the detection method, practicability and result analysis of grouting compactness of precast beam pipeline at home and abroad. However, there are certain differences between the bridge types of railway and highway Bridges, especially for the variable section beams, those difference of variable section beam thickness of the beam end and the middle part could be sizeable. The detection of conventional methods inevitably leads to detection errors, which affects the determination of detection results, and there are few studies on the detection of the density of the variable thickness beam and plate pipe. Few of the patterns that exist between test results of different plate thicknesses are involved.

Focusing on the challenge of detecting the density of prestressed pipes in bridges with varying thicknesses, this study utilized finite element software Abaqus to simulate and analyze the excitation, propagation, and reception characteristics of ultrasonic waves in concrete components. Furthermore, a theoretical time-domain analysis model was developed to detect the density of pipes containing various defects. Test results from engineering cases were concurrently compared with theoretical simulation outcomes. This study achieves integration of qualitative and quantitative relationships in ultrasonic nondestructive testing of pipeline density, thereby facilitating more convenient and rapid assessment of grouting density damage in prestressed pipelines.

## 2. The basic principle of ultrasonic compactness detection

Ultrasonic waves are a category of mechanical waves that are generated through high-frequency mechanical vibrations, typically produced by piezoelectric transducers in nondestructive testing applications. During ultrasonic wave propagation, the material discontinuity between concrete and air cavities results in significant acoustic impedance mismatches, causing partial wave reflection and energy attenuation. Structural defects in test specimens can be detected through ultrasonic signal analysis, as ultrasonic waves undergo reflection and refraction at material interfaces with different acoustic impedances. In homogeneous and defect-free materials, the ultrasonic wave propagation time exhibits a linear relationship with travel distance, while the received velocity signals provide direct characterization of the specimen’s internal structure. Consequently, ultrasonic wave velocity serves as a critical parameter for both nondestructive testing applications and defect characterization studies.

### 2.1. Wave equation of ultrasonic wave

(1) Relationship between surface wave, shear wave and longitudinal wave

Ultrasonic waves propagate on the solid surface to produce surface waves, while it propagates inside the solid to produce longitudinal and transverse waves, resulting in shear deformation and volume deformation of the medium. The relationship between longitudinal wave and shear wave can be expressed by means of auxiliary function [42], The displacement vector is the sum of the scalar function (gradient vector) and the vector function (rotation vector), which can be expressed as:


$$\vec{u}=\nabla \varphi +\nabla \vec{\varphi }$$ (1)

In the formula, φ is a scalar function of displacement field; $\vec{\varphi }$ is a vector function of the displacement field; ∇ for the Nabler symbol, $\nabla^{2}=\frac{\partial^{2}}{\partial x^{2}}+\frac{\partial^{2}}{\partial y^{2}}+\frac{\partial^{2}}{\partial z^{2}}$.

It is assumed that the concrete structure is an isotropic medium, and the displacement vector at any point satisfies:


$$\rho \frac{\partial^{2}\vec{u}}{\partial t^{2}}=\left(\lambda +\mu \right)\nabla \left(\nabla \vec{u}\right)+\mu \nabla^{2}\vec{u}$$ (2)

λ for material flexibility, μ is Poisson’s ratio for material; ρ is material density (kg/ m^3^). Replace formula (1) with formula (2), namely:


$$\rho \frac{\partial^{2}}{\partial t^{2}}\left(\nabla \varphi +\nabla \vec{\varphi }\right)=\left(\lambda +\mu \right)\nabla \left(\nabla \nabla \varphi +\nabla \nabla \vec{\varphi }\right)+\mu \nabla^{2}\left(\nabla \varphi +\nabla \vec{\varphi }\right)$$ (3)

$$\rho \frac{\partial^{2}}{\partial t^{2}}\left(\nabla \varphi +\nabla \vec{\varphi }\right)=\left(\lambda +2\mu \right)\nabla \nabla^{2}\varphi +\mu \nabla^{2}\nabla \vec{\varphi }$$ (4)

$$\nabla \times \left[\rho \frac{\partial^{2}}{\partial t^{2}}\varphi -\left(\lambda +2\mu \right)\nabla^{2}\varphi \right]+\nabla \times \left(\rho \frac{\partial^{2}}{\partial t^{2}}\vec{\varphi }-\mu \nabla^{2}\vec{\varphi }\right)=0$$ (5)

The relation between displacement field scalar and vector can be obtained by formula (5)


$$\rho \frac{\partial^{2}}{\partial t^{2}}\varphi =\left(\lambda +2\mu \right)\nabla^{2}\varphi$$ (6)

$$\rho \frac{\partial^{2}}{\partial t^{2}}=\vec{\varphi }=\mu \nabla^{2}\vec{\varphi }$$ (7)

Displacement vector of $\vec{u}$ ultrasonic field can be obtained by substituting formula (6) and formula (7) into formula (1)


$$V_{d}=\sqrt{\frac{\lambda +2\mu }{\rho }}=\sqrt{\frac{E}{\rho }\frac{1-\mu }{\left(1+\mu \right)\left(1-2\mu \right)}}$$ (8)

$$V_{p}=\sqrt{\frac{\mu }{\rho }}=\sqrt{\frac{E}{\rho }\frac{1}{2\left(1+\mu \right)}}$$ (9)

The dynamic parameter R of solid materials is introduced to represent longitudinal waves in isotropic materials $V_{d}$ and shear wave $V_{p}$ ratio available


$$R=\frac{V_{d}}{V_{p}}=\sqrt{\frac{\lambda +2\mu }{\mu }}=\sqrt{\frac{2\left(1-\mu \right)}{1-2\mu }}$$ (10)

That is:$V_{d}>V_{p}$, Hunter believes that in the same medium, shear waves and surface waves have the following relationship:


$$\frac{V_{p}}{V_{s}}=\frac{1+\mu }{0.87+1.12\mu }$$ (11)

Contrast surface waves. From formula (10) and formula (11) Longitudinal wave > shear wave > surface wave.

(2) Relationship between wave velocity and Poisson’s ratio

As evidenced by experimental studies, the propagation velocities of longitudinal, shear, and Rayleigh surface waves in bridge nondestructive testing exhibit minimal dependence on frequency, but are primarily governed by the elastic properties of the medium – specifically Young’s modulus, Poisson’s ratio, and material density [43]. Within the Poisson’s ratio range of 0.2 to 0.3 characteristic of concrete materials, the longitudinal wave velocity substantially exceeds those of shear and surface waves, with this disparity becoming more pronounced at higher Poisson’s ratios.

**Fig 1:**
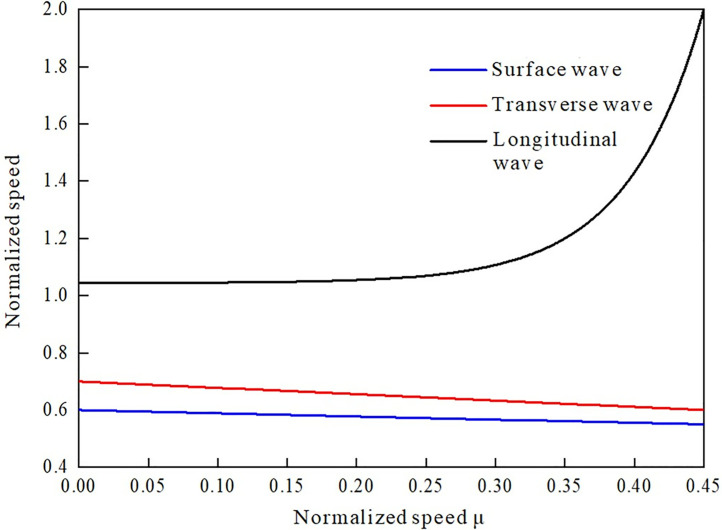
Curve of relationship between wave speed and Poisson’s ratio.

Theoretical analysis demonstrates that the velocity disparity among Rayleigh surface waves, shear waves, and longitudinal waves exhibits a positive correlation with increasing Poisson’s ratio of the material. Since this paper intends to judge compactness by comparing the first wave sound (the time when the first wave reaches the opposite side), the longitudinal wave with the largest propagation velocity is selected as the research object in the process of simulating ultrasonic excitation, propagation and reception, and the surface wave and shear wave are ignored. Under the same density and different Poisson ratios, the distribution of each wave velocity is shown in Fig 1 [44].

### 2.2. Excitation and reception of ultrasonic wave

(1) Ultrasonic excitation

For pipeline grouting density assessment, the ultrasonic testing methodology employs electromagnetic acoustic transducers to generate excitation signals through controlled mechanical impulses. These induced ultrasonic waves then propagate through the structural member along predetermined wave paths. The frequency and period of the excitation point are the same as the frequency and period of the ultrasonic wave. The relationship between the ultrasonic wave velocity, frequency and wavelength is


$$\lambda =\frac{c}{f}$$ (12)

(2) Ultrasonic propagation

For a single concrete medium, the longitudinal wave propagation velocity remains unchanged; when the bonding between the beam and the pipeline grouting slurry is dense, and the thickness of the beam plate is determined, the first wave sound of the sound wave is fixed. As evidenced by the analytical results, the first-arrival time difference, signal amplitude variation, and phase shift characteristics exhibit distinct patterns corresponding to varying degrees of pipeline grouting compaction. These ultrasonic parameters serve as critical indicators for both compaction quality assessment and underlying mechanical interaction analysis.

(3) Ultrasonic testing process

**Fig 2:**
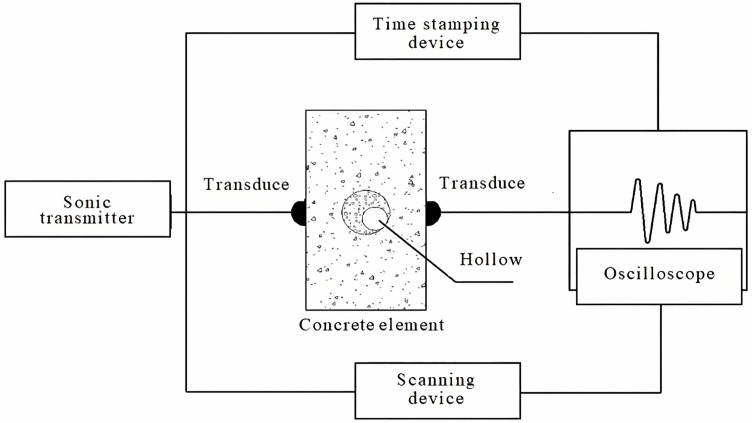
Schematic diagram of ultrasonic method.

Ultrasonic emission and receiving probes were positioned on opposite sides of the tested component, aligned perpendicular to the pipeline’s center. Coupling agents, such as grease, were applied to ensure close contact between the probes and the component surface. Waveform measurement is performed by amplifying the received signal, while a time-scaling device analyzes ultrasonic time, phase, and other related information. The principle of ultrasonic measurement is shown in Fig 2.

(4) Matters needing attention in ultrasonic testing

When using ultrasonic testing method to detect the density of prefabricated beam, attention should be paid: ① Ensure that both sides of the tested member have test operation space; ② Before detection, the probe should be calibrated and the position should be determined; ③ The ultrasonic transmitter and the signal receiver are placed on the same plane to keep the two positive.

When there are holes or cracks in the prestressed pipe, because the impedance of concrete is much greater than that of air, ultrasonic wave will diffraction and reflection at the interface between the two, increasing the propagation distance, weakening the energy of ultrasonic wave, the amplitude of the received signal decreases, the frequency decreases [45], and the first wave increases. Comparative analysis of ultrasonic dynamic and kinematic parameters between fully-grouted ducts and void-containing sections enables precise spatial identification of grouting defects, including both void localization and volumetric extent quantification.

## 3. Theoretical model of ultrasonic testing density of variable thickness plate

If the medium and plate thickness of the ultrasonic wave can be determined, it can be calculated by calculating the difference between the first wave acoustic time and the acoustic time after the cavity with a given plate thickness. On the contrary, the compactness of concrete slab can be calculated according to the first wave sound. Basis on this train of thought, the quantitative and qualitative theoretical relationship between the first wave sound time and the grouting compactness of prestressed pipeline is further studied in this paper.

### 3.1. The basic assumption of the model

Based on ensuring sufficient accuracy and accuracy, considering the actual situation of the project, based on the principle of theoretical model simplification, the following basic assumptions are needed:

1) Assuming that the boundary between each unit is continuous;
2) The theoretical model is an explicit dynamic problem without considering the influence of gravity;
3) Only considering the impact load and in the linear elastic state, the displacement, stress and strain of each particle obey Hooke’s law;
4) Concrete is isotropic material.

### 3.2. Parameter analysis and selection of theoretical model

In the process of beam propagation, when the steel strand is selected as the ultrasonic transmission medium, the energy of the steel strand will be reduced, but the ultrasonic wave propagates faster in the steel strand. The ultrasonic propagation path around the steel strand will become longer, but the impedance of the steel strand is less than that of the concrete, and the wave velocity increases. The two ultrasonic waves inside and on the surface of the strand converge below the strand; therefore, the existence of steel strands in the pipeline has little effect on the ultrasonic signal. However, in order to improve the accuracy of the model and reflect the reliable data, the metal steel strand is set according to the construction requirements. In this paper, the project is based on the prestressed pipe formed by rubber drawing, so the metal bellows are not considered. The physical parameters of the material in the model are shown in Table 1.

**Table 1:** Concrete physical parameters.

Concrete	Density ρ(kg/m^3^)	Modulus of elasticity E(GPa)	Poisson’s ratio(μ)
C50	2500	34	0.25

### 3.3. Application of ultrasonic load

An impact load is applied at the excitation point to simulate the vibrator’s vibration for emitting ultrasonic waves. The theoretical model established for ultrasonic detection represents a high-speed dynamic problem. The impact load and excitation time at the ultrasonic source can be approximated by a half-period sine function [46]. The impact load at different times can be expressed as


$$F\left(t\right)=\left\{\;\;\;\begin{array}{c} {}*20cF_{\max}\sin\left(\frac{\pi }{T}t\right),0\leq t\leq T_{c}\\ 0,t\geq T_{c} \end{array}\;\;\;\right\}$$ (13)

Since the load does not affect the frequency of ultrasonic signal, in order to make the theoretical simulation results more reliable, it should be increased as much as possible (the maximum impact load is 100 N). In the finite element simulation of acoustic characteristics, it is required that the ultrasonic propagation distance should not exceed two times of the thickness of the measured object within the duration of the impact load. The duration of the impact load is only related to the ultrasonic wave velocity and the thickness of the beam and plate, namely, $t_{c}<\frac{2D}{V_{d}}$

Calculation can be obtained, reference $t_{c}<\frac{2D}{V_{d}}=\frac{2\times 40}{4040}=198\mu s$ values, this paper set the impact load duration of 30μs [31].

**Fig 3:**
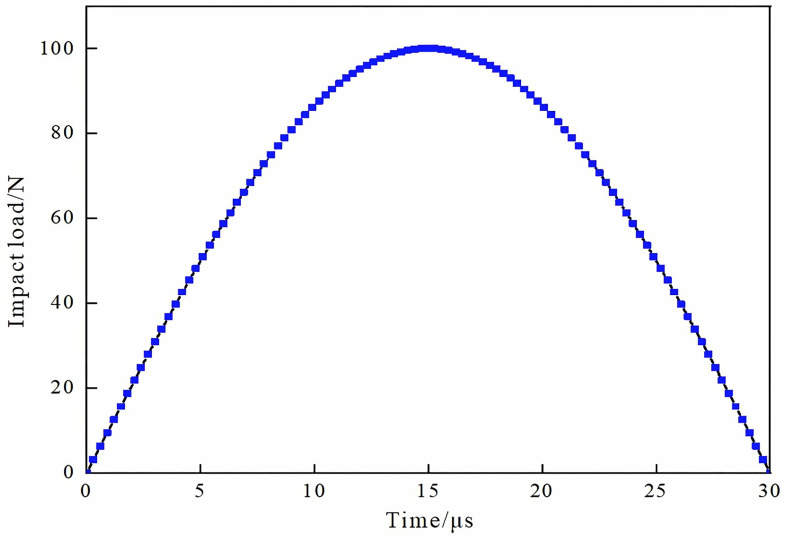
Impact load amplitude.

The amplitude curve of the impact load and the duration of the simulated ultrasonic excitation are shown in Fig 3.

### 3.4. Model grid unit division

Under the action of impact load, the longitudinal wave signal is selected as the research object. When dividing the unit size, it is necessary to satisfy that the divided unit can capture the output of the longitudinal wave propagation field. The maximum unit size of the theoretical model grid of ultrasonic propagation characteristics should meet


$$L{\frac{\lambda_{\min}}{20}}_{\max}$$ (14)

In C50 concrete, the longitudinal wave velocity reaches 4040 m/s, with an effective frequency range of 30–40 kHz and a minimum effective wavelength of approximately 130 mm. Accordingly, the maximum grid unit size should range from 6 mm to 8 mm. To further ensure accuracy, the element size in the theoretical model is ultimately set to 5 mm.

### 3.5. Results and laws of theoretical model

**Fig 4:**
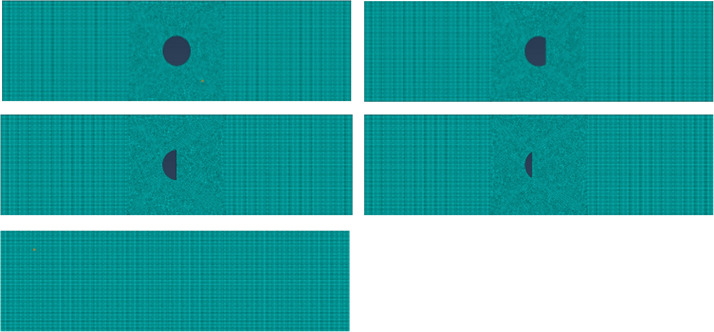
Theoretical model of 40 cm slab thickness concrete. (a) All empty; (b) 0.25 the compactness; (c) 0.5 the compactness; (d) 0.75 the compactness; (e) 1 the compactness.

In view of the small size of the element mesh and the small difference between the longitudinal sections of the component, the two-dimensional solid model can meet the research needs. Seven concrete slab models with sections of 0.4 m × 1.5 m, 0.5 m × 1.5 m, 0.6 m × 1.5 m, 0.7 m × 1.5 m, 0.8 m × 1.5 m, 0.9 m × 1.5 m and 1.0 m × 1.5 m are established. Prestressed pipes and steel strands of various specifications are positioned at the centroid of the actual structure to verify the validity of the theoretical analysis. Select C50 concrete, pipe diameter of 12 cm, 0.4 m × 1.5 m size model, mesh size of 5 mm, in the upper side of the beam and plate center and lower center set ultrasonic transmitting point and ultrasonic receiving point, the impact load of the transmitting point is set according to the formula (13), the extreme load is 100 N, the theoretical model is established as shown in Fig 4.

**Fig 5:**
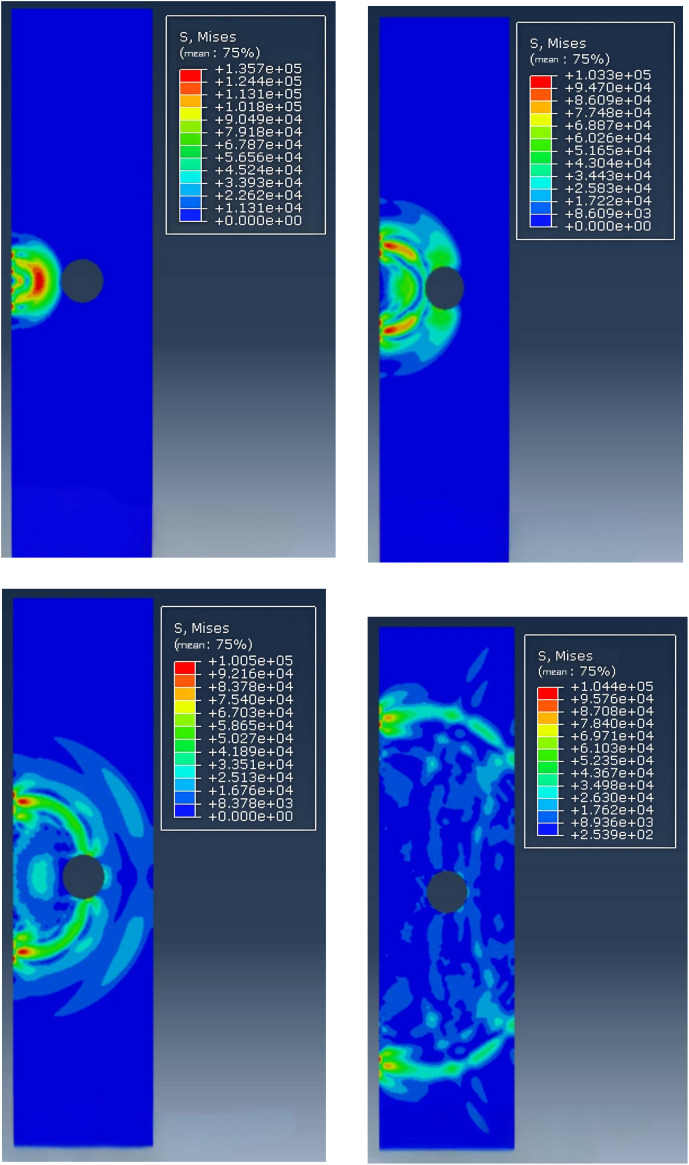
Stress-strain cloud diagram at various moments.

The stress-strain nephogram of each time can be obtained by theoretical model calculation as shown in Fig 5. Analysis of stress nephogram at each moment shows:

(1) At 39.10 μs, ultrasonic waves arrive at the prestressed pipe and reflect and diffraction occurs on the surface of the pipe;
(2) At 70.99μs, the reflection wave on the top surface of the prestressed pipe reaches the top of the beam plate;
(3) At 111.25μs, the incident wave passes through the prestressed pipe to the bottom of the beam plate;
(4) At 250.80μs, the reflection wave at the bottom of the beam plate reaches the top of the beam plate;
(5) A small part of the ultrasonic wave will reflect and propagate to the roof when encountering the pipeline, and the other part will diffraction and propagate to the bottom plate through the pipeline, and then reflect and propagate to the roof. The propagation path of ultrasonic wave in concrete beams and slabs is obvious.
(6) The time from the emission point to the receiving point of the ultrasonic wave of the fully dense concrete slab is 102.01μs (the longitudinal wave velocity is 4040 m/ s). The thickness of the beam slab is 0.4121m, which is close to the thickness of 0.4m, further verifying the reliability and accuracy of the theoretical model.

## 4. Determination of first wave acoustic time—Density relation

In order to further study the law between the density of pipes with different plate thicknesses and the first wave sound time based on the above theoretical model. Dozens of models have been established as follows: the concrete slab structure with the thickness of 40 cm ~ 100 cm and the pipe diameter of 12 cm was selected. Considering the five working conditions of full space, 0.25 compactness, 0.5 compactness, 0.75 compactness and full compactness, a total of 35 beam-slab models were established, and all the time-domain diagrams of different slab thickness models were obtained. Ultimately, an intrinsic constitutive relationship is established between the first-wave acoustic time across different plate thicknesses and the grouting density.

**Fig 6:**
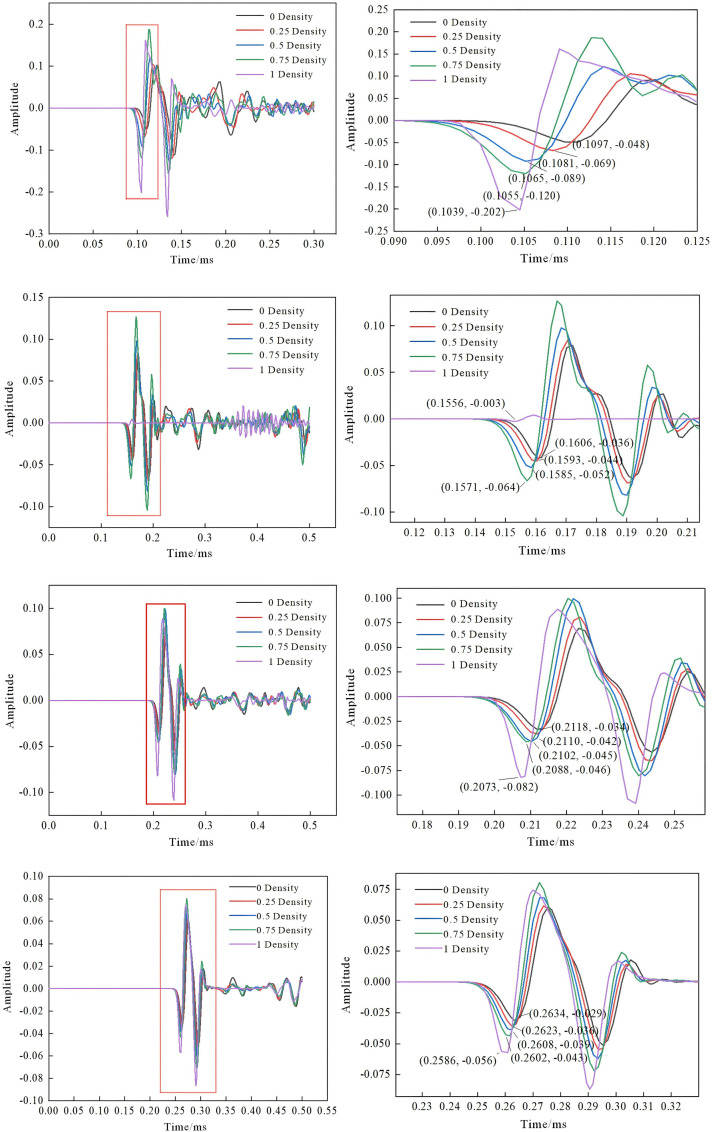
Time domain diagram and partial enlarged diagram of each plate. (a) Time domain and local diagrams of 40 cm plate thickness; (b) Time domain and local diagrams of 60 cm plate thickness; (c) Time domain and local diagrams of 80 cm plate thickness; (d) Time domain and local diagrams of 100 cm plate thickness.

The time domain analysis and local amplification of the theoretical model are shown in Fig 6.

Analysis of the time-domain diagrams under five working conditions reveals that, with a constant pipe diameter, the time-domain curves vary across different plate thicknesses, and the first-wave arrival time increases as the plate thickness increases. Under the same plate thickness, the first wave acoustic time decreases with the increase of density. The first sound time values for each plate thickness are summarized in Table 2.

**Table 2:** Board thickness-first wave acoustic time data.

CompactnessThickness (cm)	0	0.25	0.5	0.75	1
40	109.7	108.1	106.5	105.5	103.9
50	135.1	133.8	132.3	131.0	129.6
60	160.6	159.3	158.5	157.1	155.6
70	186.7	185.2	185.1	183.5	182.1
80	211.8	211.0	210.2	208.8	207.3
90	238.4	236.9	236.9	235.4	233.5
100	263.4	262.3	260.8	260.2	258.6

Based on the obtained data, it is found that the smaller the duration value of the first wave of sound, the greater the corresponding density. In order to quantitatively obtain the law between the first wave acoustic time and the density, and to facilitate the discovery of its correlation, the first wave acoustic time with defects and the first wave acoustic time with complete density are quotient to try to summarize the law of (t defect/ t density) −1. Calculation results are presented in Table 3. Here, t_defect represents the first-wave acoustic time corresponding to a density range of 0 to 0.75, while t_density denotes the first-wave acoustic time at full density.

**Table 3:** (T _defect_/t _density_) −1 data.

CompactnessThickness (cm)	0	0.25	0.5	0.75	1
40	0.056	0.040	0.025	0.015	0.000
50	0.042	0.032	0.021	0.011	0.000
60	0.032	0.024	0.019	0.010	0.000
70	0.025	0.017	0.016	0.008	0.000
80	0.022	0.018	0.014	0.007	0.000
90	0.021	0.015	0.015	0.008	0.000
100	0.019	0.014	0.009	0.006	0.000

**Fig 7:**
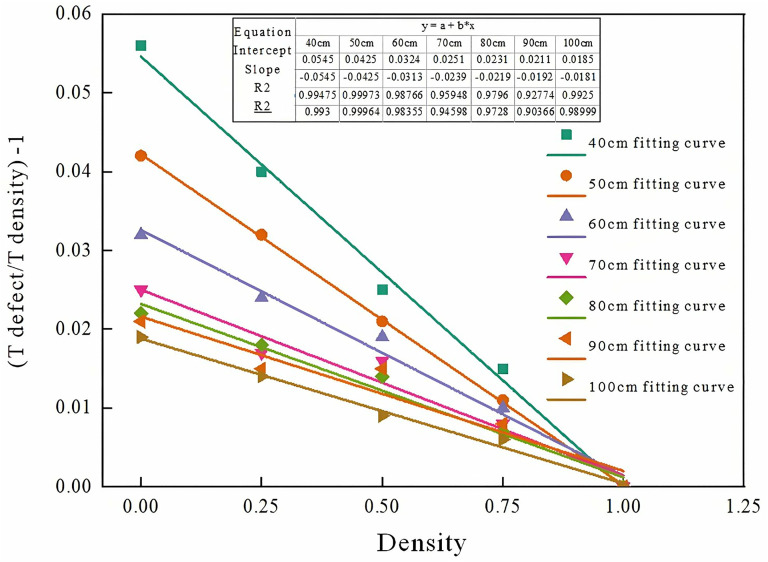
Fitting curve of first wave acoustic time for different plate thickness models.

Numerical fitting between (t defect/ t density) − 1and compactness for eight beam-plate models with varying thicknesses is presented in Fig 7. Slopes and intercepts of the fitting curves for each plate thickness are summarized in Table 4.

**Table 4:** The slope of the fitting curve of the first wave of each plate thickness model.

Thickness	The slope	Intercept	Fitted Curve Regression
40 cm	−0.0545	0.0545	y = 0.0545−0.0545x
50 cm	−0.0425	0.0425	y = 0.0425−0.0425x
60 cm	−0.0313	0.0324	y = 0.0324−0.0313x
70 cm	−0.0239	0.0251	y = 0.0251−0.0239x
80 cm	−0.0219	0.0231	y = 0.0231−0.0219x
90 cm	−0.0192	0.0211	y = 0.0211−0.0192x
100 cm	−0.0181	0.0185	y = 0.0185−0.0181x

**Fig 8:**
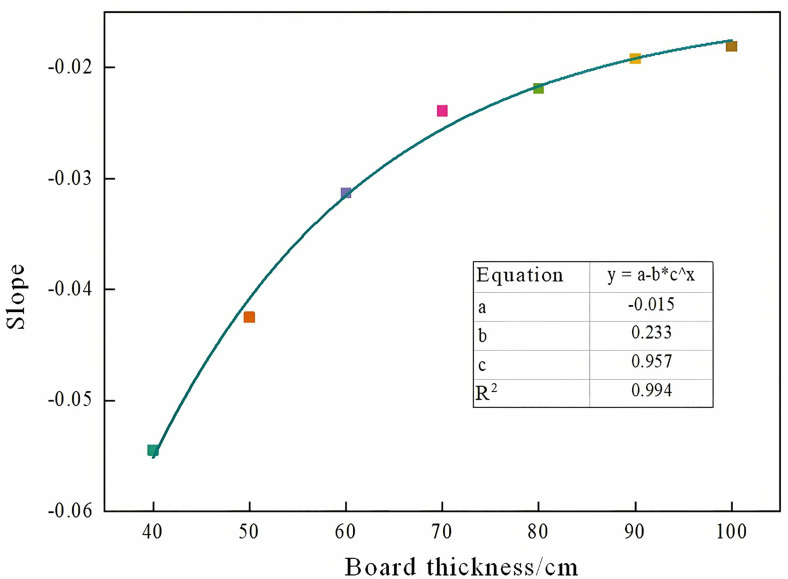
The trend of the slope of different plate thicknesses.

**Fig 9:**
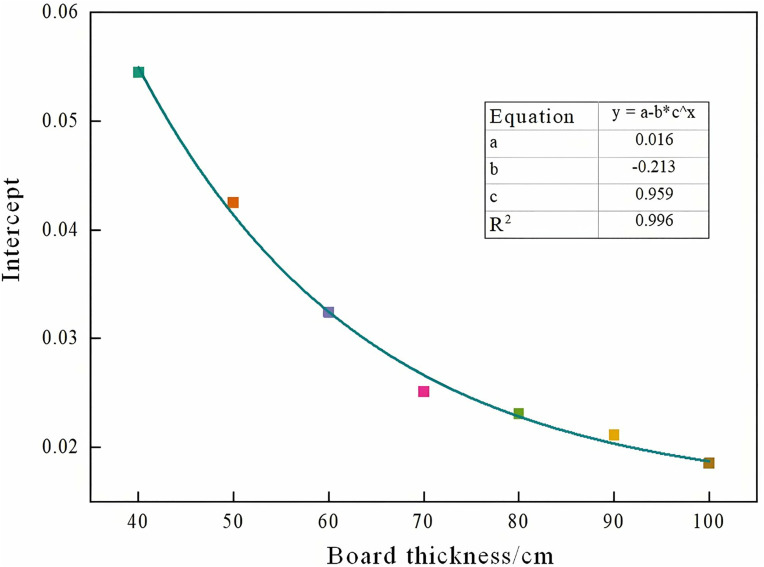
Variation trend of intercepts of different plate thicknesses.

Analysis of Table 4 and Fig 7 indicates that both the slope and intercept of the first-wave fitting curves for models with varying plate thicknesses exhibit a clear asymptotic relationship with beam and plate thickness. Variation trends of slope and intercept across different plate thicknesses are illustrated in Figs 8 and 9. Further fitting of these parameters yielded a slope of [please insert value], an intercept of [please insert value], and a coefficient of determination (COD) exceeding 0.99, demonstrating excellent fitting accuracy.

By substituting the slope and intercept into the fitting curve formula of the pipeline, the function expression between the first wave acoustic time, the thickness of the beam and the grouting density can be obtained.


$$Z=\left(-0.015-0.233\times 0.957^{d}\right)C+\left(0.016+0.213\times 0.959^{d}\right)$$ (15)

The formula is (t defect/ t dense) −1, when t defect and t dense are 0 ~ 1 dense, the first wave acoustic time value (μs); *d* is the thickness of the measured member plate, *C* for grouting density.

## 5. Application example verification of pipeline density detection

### 5.1. Project profile

**Fig 10:**
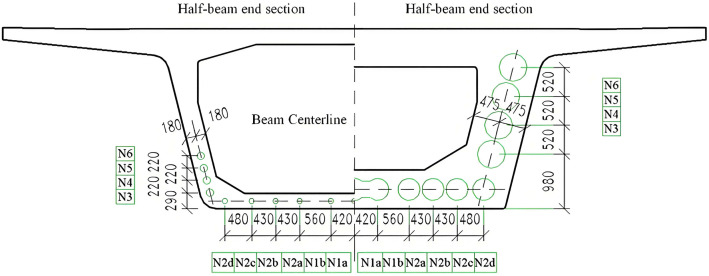
Cross section of a beam bridge of a high-speed railway.

In order to tested accuracy of the theoretical model, the high-speed railway and highway girder bridge are selected as the test objects, and the pipe density of prestressed bridges with different plate thicknesses is tested non-destructive detection. The quantitative and qualitative relationship between the first acoustic time and the density can also been verified in the process. Among them, the cross section of 1/ 2 span and 1/ 2 beam end of high-speed railway bridge is shown in Fig 10. Its structural form is a single box 40 m span prefabricated box girder, the minimum plate thickness is 36 cm, and the maximum plate thickness is 95 cm.

**Fig 11:**
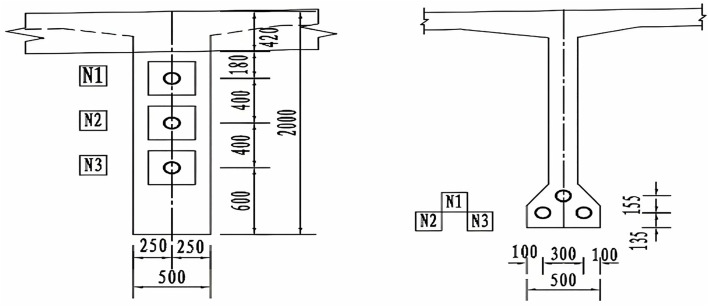
Cross-section of a beam bridge of an expressway.

Fig 11 illustrates the layout of prestressed steel strands in the cross section of a highway bridge. A precast T-beam with a 30 m span is used, featuring a web thickness of 50 cm at the beam ends and 30 cm at mid-span.

### 5.2. Detection method and measured results

(1) Qualitative detection

Before positioning detection, it is necessary to qualitatively detect the whole bridge to obtain the general situation of grouting quality of each pipeline. Because the grouting compactness of the beam ends of the pipelines on both sides of large mileage and small mileage is different, it is necessary to detect the two ends of the same pipeline, to make the qualitative detection results more accurate and reasonable.

(2) Qualitative test results

Field qualitative detection results indicate that the grouting compactness of selected N5 and N6 pipelines in the right section of the high-speed railway bridge, as well as N1 and N2 pipelines in the highway bridge, is relatively low and fails to meet the standard requirement of a comprehensive grouting index exceeding 98%. Further positioning detection is required to accurately locate the cavities.

(3) Location detection

**Fig 12:**
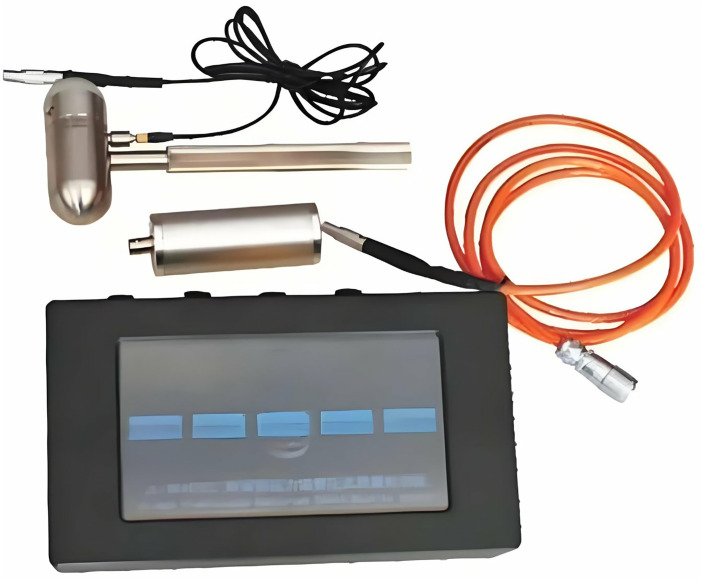
JL-BPAC bridge prestressed hole grouting quality detector.

**Fig 13:**
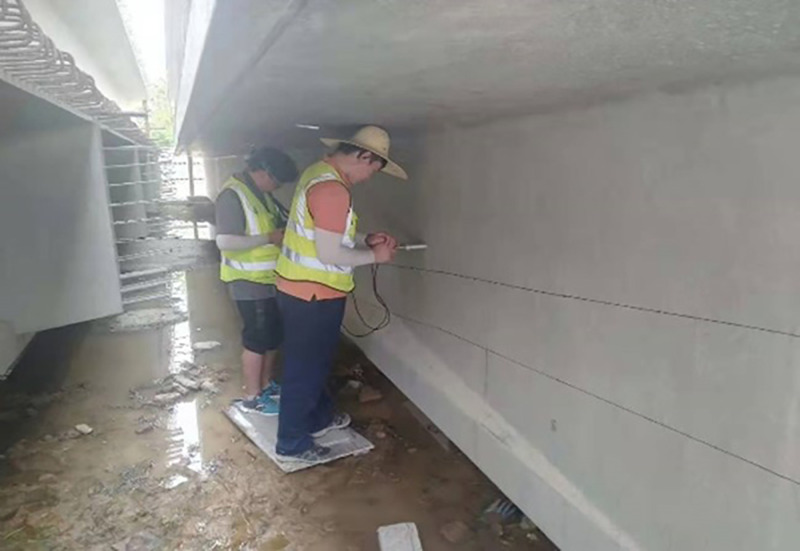
Distribution of survey lines and inspection photos.

Pipelines exhibiting a low mass index were selected for ultrasonic positioning detection. The JL-BPAC bridge prestressed pipeline grouting quality detector, shown in Fig 12, was employed as the detection instrument. Measuring points were arranged sequentially at 20 cm intervals from the pipeline end along its length. Each measurement point was ensured to have a clean, smooth surface free of loose soil; any uneven pits along the pipeline were polished prior to measurement. Distribution of measuring lines and detection photographs are presented in Fig 13.

(4) Location detection results

**Fig 14:**
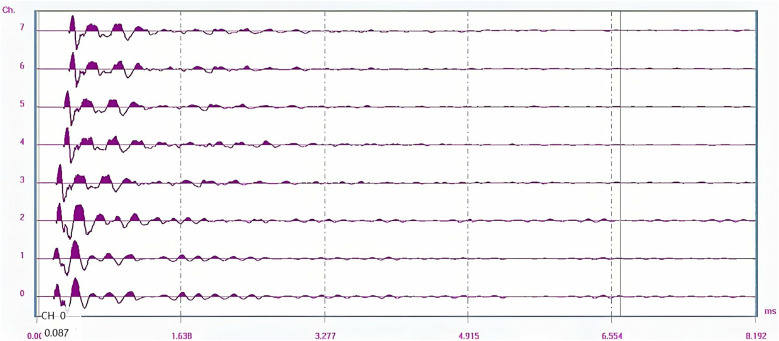
The measured compactness.

To verify the accuracy of the theoretical formula relating first-wave acoustic time to density, measurements at 95 cm, 80 cm, 50 cm, and 35 cm points were specifically recorded along with their corresponding first-wave acoustic times. The associated test waveform is presented in Fig 14, and the measured data are summarized in Table 5.

**Table 5:** Measured data.

Pipe number	Thickness	The first wave sound	Measured compactness
High speed railway N6	95 cm	246.71	89.6%
High speed railway N5	95 cm	246.52	94.2%
High speed railway N6	80 cm	207.79	88.1%
High speed railway N5	80 cm	207.51	96.8%
Highway N1	50 cm	129.89	93.6%
Highway N2	50 cm	129.64	96.1%
Highway N1	35 cm	87.08	95.6%
Highway N2	35 cm	87.00	97.1%

### 5.3. Comparative analysis of theoretical results and measured data

Two measuring points of 95 cm N6 pipeline plate thickness of high-speed railway bridge and 50 cm N1 pipeline plate thickness of highway bridge is selected as examples to replace the theoretical formula, that is, the plate thickness and the first acoustic age are introduced into the formula (15), namely


$$246.71=\left(-0.015-0.233\times 0.957^{95}\right)C+\left(0.016+0.213\times 0.959^{95}\right),C_{95}=0.92$$

$$129.89=\left(-0.015-0.233\times 0.957^{50}\right)C+\left(0.0156+0.213\times 0.959^{50}\right),C_{50}=0.96$$

**Fig 15:**
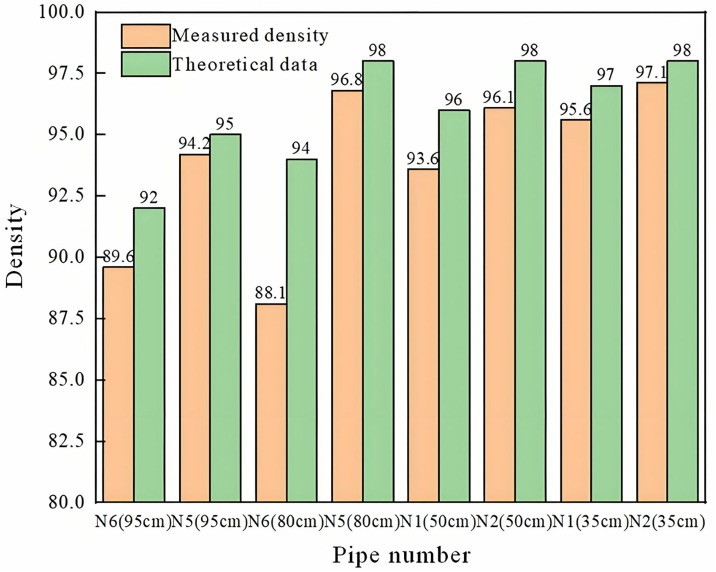
Comparison chart of theoretical and measured results.

The compactness calculated by the theoretical formula is compared with the measured results, and the summary is shown in Fig 15.

It can be seen from Fig 15 that:

(1) Results calculated using the time-domain analysis formula show good agreement with field measurements;
(2) Theoretical calculations slightly overestimate the measured results, primarily due to various environmental factors affecting the actual measurement process. Misalignment among the ultrasonic excitation point, signal receiving point, and pipeline—causing deviation from the intended measurement plane—leads to discrepancies in detection location. Consequently, the cavity position deviates from reality, the measured cavity size appears smaller, and the resulting density values are higher;

(3) For pipelines with high measured compactness such as N5 (80 cm), N2 (50 cm) and N1 (35 cm), the error between theoretical calculation results and measured data can be guaranteed within 5%.

It can be seen from the analysis that the error between the calculated results and the measured compactness is within 10%, and it is feasible to determine the pipeline grouting compactness by the time domain analysis method based on the law between the plate thickness and the first wave sound time. According to the results of on-site windowing test, there is a phenomenon of grout shedding from the pipeline of high-speed railway bridge N6 and N5 and highway bridge N1 and N2 within a certain range, which further verifies the correctness of the theoretical relationship between the first wave sound time and compactness.

## 6. Conclusion

Based on the ultrasonic detection method, this research deeply studied the basic principle of ultrasonic defect detection and established a theoretical model for ultrasonic detection of variable section beam. Theoretical analysis systematically established the relationship between first-wave acoustic time and compactness in ultrasonic testing of prefabricated beams with variable thickness. This study creatively proposed and validated the correlation between quantitatively measured beam compactness in the field and the corresponding qualitative theory.

(1) Using the auxiliary function, the magnitude relationship between the longitudinal wave and the transverse wave in the solid medium is deduced through the relationship between the displacement field scalar function and the vector function. The results show that with the increase of Poisson’s ratio μ, the wave velocity of longitudinal wave is significantly greater than that of transverse wave. Therefore, longitudinal waves should be the focus of attention when studying the first wave of ultrasonic waves in solids.
(2) Steel strands have minimal influence on ultrasonic signals and can be neglected to simplify calculations when developing the theoretical model. Impact load magnitude does not affect the acoustic signal frequency, and accuracy can be ensured by setting the maximum impact load as high as possible. The duration is only related to the propagation distance and wave speed, and it is enough to satisfy $t_{c}<\frac{2D}{V_{d}}$; The minimum mesh size of the model is related to the wavelength of the ultrasonic wave. In the case of satisfying the accuracy, an appropriate mesh size should be selected for the calculation.
(3) Ultrasonic waves are reflected and scattered upon reaching the pipe surface during propagation, leading to an increase in the first-wave acoustic time; The research shows that the theoretical relationship between the first wave acoustic time value(Z), the thickness of the beam plate(d) and the grouting compactness(C) is that $Z=\left(-0.015-0.233\times 0.957^{d}\right)C+\left(0.016+0.213\times 0.959^{d}\right)$.
(4) Using ultrasonic testing to evaluate pipeline compactness enables the identification and sizing of internal defects by analyzing first-wave acoustic data. The calculated results tend to overestimate actual measurements; however, with precise positioning of measurement points, the error can be maintained within 10%, ensuring relatively high reliability.
(5) The research results can provide theoretical support for bridge pipeline compactness detection engineering.

### Notation

The following symbols are used in this paper

**Table:** 

ϕ=Scalar function of displacement field;
$\overset{\to }{\phi }$=Vector function of the displacement field;
$\vec{\text{u}}$=Displacement vector of ultrasonic field;
∇=Nabler symbol;
ρ=Density;
*λ* = Wavelength of ultrasonic wave;
*μ* = Poisson’s ratio;
${\text{V}}_{\text{d}}$=Transverse wave;
${\text{V}}_{\text{p}}$=Longitudinal wave;
${\text{V}}_{\text{s}}$=Surface wave;
R=Ratio of transverse wave to longitudinal wave;
c=Ultrasonic wave velocity;
f=the ultrasonic frequency;
${\text{T}}_{\text{c}}$=impact load duration;
${\text{F}}_{\text{max}}$=maximum impact load;
E=Elastic Modulus;
D=Concrete beam thickness;
${\text{L}}_{\text{man}}$=Minimum cell size of the grid;
$\lambda_{\text{min}}$=Minimum wavelength;
Z=(T _defect_/T _density_) -1;
C=Grouting compactness.
